# Comparison of incisional complications between skin closures using a simple continuous or intradermal pattern: a pilot study in horses undergoing ventral median celiotomy

**DOI:** 10.7717/peerj.5772

**Published:** 2018-11-09

**Authors:** Doreen Scharner, Claudia Gittel, Karsten Winter, Dominique Blaue, Carola Schedlbauer, Ingrid Vervuert, Walter Brehm

**Affiliations:** 1 Department for Horses, University of Leipzig, Leipzig, Germany; 2 Institute of Anatomy, Faculty of Medicine, University of Leipzig, Leipzig, Germany; 3 Institute of Animal Nutrition, Nutrition Diseases and Dietetics, University of Leipzig, Leipzig, Germany

**Keywords:** Exploratory laparotomy, Surgical site infection, Suture pattern, Complications, Incision

## Abstract

**Background:**

Development of incisional complications following ventral median celiotomy might depend on suture pattern for skin closure.

**Methods:**

In this prospective study, 21 healthy male horses underwent celiotomy. Skin closure was either performed via a continuous percutaneous pattern (CO group; 5 warmbloods/5 ponies) or an intradermal pattern (ID group; 5 warmbloods/6 ponies). Follow-up examination of the incisional site included daily monitoring for edema, dehiscence, and drainage. Transcutaneous ultrasound was performed at Days 3, 6, and 10 as well as on Week 8 and 12 to evaluate size of edema and presence or absence of sinus formation, and hernia formation. Prevalence of incisional infection on base of positive microbiological analysis at any time up to Day 10 was evaluated and compared between ID and CO group. Furthermore, edema size was analysed by a linear mixed-effect model for group and time dependency.

**Results:**

Observed incisional complications included edema (9/10 in CO, 10/11 in ID), suture sinus formation (2/10 in CO, 1/11 in ID), surgical site infection (2/10 in CO, 0/11 in ID), and incisional hernia (1/10 in CO, 0/11 in ID). The overall prevalence of incisional infection was 9.5% without significant differences between both groups (20% in CO, 0% in ID; *p* = 0.214). Edema size was not dependent on time or group (*p* = 0.545 and *p* = 0.627, respectively).

**Discussion:**

CO and ID suture pattern are appropriate for skin closure following ventral median celiotomy in horses. None of the animals in the continuous ID group developed surgical site infections, even without the use of antibiotics.

## Introduction

Incisional complications, including edema, dehiscence, drainage or surgical site infection, and incisional hernia occur commonly following ventral midline celiotomy in horses, leading to prolonged hospitalization, longer recovery times and increased cost (Kobluk et al., 1989; French et al., 2002; Mair & Smith, 2005). Despite ongoing research and advances in surgical technique over the last decade, rates cited for incisional complications still range from 16% to 62% (Lippold, 2001; Proudman et al., 2002; Mair & Smith, 2005; Freeman et al., 2012).

Many factors have been investigated for their influence on development of incisional complications, for example, breed, sex and pre-surgical condition of the horse (Colbath et al., 2014; Darnaud et al., 2016). However, they are fixed and can rarely be modified by the surgeon.

Several studies have focused on identifying risk factors associated with assorted suture materials and closure patterns. Factors that predispose to incisional complications include a near-far-far-near pattern (Kobluk et al., 1989) and use of chromic catgut (Gibson et al., 1989) for closure of linea alba. In one retrospective study, greater risk of incisional infection was evident with use of polyglaction-910 (vs. polydioxonone) suture (Honnas & Cohen, 1997). On the other hand, antibacterial coating of suture appears ineffective as a preventive measure in this setting (Bischofberger et al., 2010). In another study, the risk of incisional infection was increased by stapling of skin incisions (Torfs et al., 2010). Colbath et al. (2014) showed a reduction of incisional drainage with the use of a two-layer, modified subcuticular closure. However, until now, no clear consensus has emerged on a type of suture or a suturing pattern that is optimal to avoid complications in abdominal incisions (Salem, Proudman & Archer, 2016). Whether an intradermal, full-thickness continuous or an interrupted pattern is the best skin suture pattern to prevent surgical site infection in closure of ventral median celiotomy in horses is not apparent and further investigation is warranted (Torfs et al., 2010).

The objective of this study was to compare incisional healing complications between skin closures using a simple continuous (CO) or intradermal (ID) pattern in horses undergoing celiotomy. Based on our clinical impression, we hypothesized, that ID suturing might lower the risk of incisional complications.

## Materials and Methods

This prospective study focusing on skin suture pattern was a spin-off of a larger project (approved by the local ethics committee, Landesdirektion Leipzig, TVV 32/15), in which 10 warmbloods and 11 ponies underwent celiotomy for harvest of abdominal fat and liver tissue. The animals were housed in individual straw-bedded boxes and were fed a hay diet. In advance, ponies and horses, evenly distributed, were selected at random for assignment of different skin suture pattern (simple continuous (CO) or intradermal (ID) group) by drawing lots.

All individuals were subjected to a lipopolysaccharide (LPS) challenge 15 h before surgery as part of the main project. For this purpose, LPS (10 ng/kg) from *Escherichia coli* 055:B5^1^1L6529, Lot Number 124M4D49V, Sigma-Aldrich Chemie GmbH, Munich, Germany. was administered IV over 30 min. No analgesic was given prior to surgery.

In preparation for surgery, food but not water was withheld overnight. A standardized anesthesia protocol was used and consisted of IV 0.08 mg/kg romidifine and 0.03 m/kg butorphanol for premedication, followed 10 min later by IV 0.08 mg/kg diazepam and 3 mg/kg ketamine for induction. For maintenance of anesthesia, animals were connected to a circle breathing circuit with isoflurane in oxygen. Additionally, lidocaine (0.05 mg/kg/min) was administered IV. No systemic or local antibiotics were applied pre-, intra- or post-surgery.

For surgery, horses were positioned in dorsal recumbency and the surgical site was prepared for aseptic surgery. At first, the prepuce was covered with dry gauze and closed with towel clamps. Afterwards, the ventral abdomen was clipped, and washed with antiseptic soap (Degraseptin®; Albrecht, Aulendorf, Germany), defatted with ethanol (PKH GmbH, Halle, Germany) and disinfected with iodine and 70% ethanol (Braunoderm®; B. Braun Medical AG, Melsungen, Germany). None of the animals had any evidence of previous abdominal surgery. One surgeon (WB) performed all surgical procedures. A 20 cm celiotomy (length determined via sterile ruler) was made in the pre-umbilical region on ventral skin midline by incision of skin, subcutis, and linea alba and a subseqeuent sharp dissection of ligamentum teres hepatis. In case of excessive gas accumulation in the large colon and cecum, decompression was performed via a suction unit using a 20-G hypodermic needle. To enable intraabdominal manipulation the abdominal cavity was irrigated with sterile lactated Ringer’s solution (ponies, 500 ml; warmbloods, 1,000 ml). Retroperitoneal and mesocolic fat were harvested by dissection, and liver specimens were sampled via biopsy punch. In each instance, one to three g tissue was collected as part of the main study. In the event of serosal injuries, mesocolic suturing was performed at the discretion of the surgeon. Each abdominal incision was closed in four layers, whereby peritoneum, linea alba, and subcutis were closed equally in both groups (see Table 1). Skin closure was either performed via simple continuous (CO group) or via intradermal suture (ID group), with differences in suture pattern and suture material (see Table 1). Subsequent to wound closure a self-adhesive drape (Fixomull® stretch; BSN Medical GmbH, Hamburg, Germany) was placed on the midline and secured with skin staples for incisional protection. In case of any loss of the drape during recovery, this was noticed.

**Table 1:** Detailed description of suture pattern and suture material.

Sutured tissue layer	Group	Suture pattern	Suture material	Brand name	Suture size (metric)
Peritoneum	CO, ID	Simple continuous	Polyglactin 910	Coated Vicryl™ rapide^a^	3.5
Linea alba	CO, ID	Simple continuous, bite-size at about 12 mm intervals	Lactomer 9-1 loop	Polysorb™^b^	5
Subcutis	CO, ID	Simple continuous	Antibacterial polyglactin 910	Vicryl™ Plus^c^	4
Skin	CO	Simple continuous, percutaneous	Polyamide	Supramid^d^	4
	ID	Simple continuous, subcuticular	Antibacterial polyglactin 910	Vicryl™ Plus^c^	4

**Notes:**

**Skin closure:** CO, continuous percutaneous pattern; ID, intradermal pattern.

**Manufacturer**

a Johnson & Johnson Medical GmbH, Ethicon Deutschland, Norderstedt, Germany.

b Covidien Germany GmbH, Neustadt an der Donau, Germany.

c Johnson & Johnson Medical GmbH, Ethicon Deutschland, Norderstedt, Germany.

d B. Braun, Vet Care GmbH, Melsungen, Germany.

Postsurgical treatment consisted of flunixin–meglumine with an initial dose of 1.1 mg/kg IV, followed by 0.6 mg/kg twice daily for 72 h (peroral). Following surgery, animals were subjected to physical examinations, routine blood work and pain evaluations during the intensive care period of 10 days. Self-adhesive drapes were replaced at 24 h after surgery and ultrasound of the ventral abdomen was performed for absence of increased peritoneal fluid. Wound protections were removed on Day 3. In the CO group, sutures were removed on Day 10 after disinfection of skin with iodine and 70% ethandol (Braunoderm®). Box rest was maintained for 12 weeks, thereafter horses were allowed to have access to a small paddock. Postoperative examinations of the abdominal incision were terminated on Week 12.

Progress of wound healing and incisional complications were assessed in a standardized manner by one surgeon (DS), who was aware of the respective skin suture.

Short-term follow-up (Day 1–Day 10) consisted of presence or absence (yes/no) of edema, dehiscence, and drainage at the incision site by clinical examination of the surgical site once daily. Any type of incisional drainage was defined as surgical site infection (Torfs et al., 2010; Freeman et al., 2012; Colbath et al., 2014). Additionally, ultrasound of the surgical site was performed on Days 3, 6, and 10. To avoid contamination of the incision, the probe was covered with sterile surgical probes and sterile ultrasound gel was used (Sonogel®; Sonogel, Bad Camberg, Germany). Subcutaneous edema size immediately adjacent to the incision site was recorded at three locations (cranial, centre and caudal part of the incision). Post hoc, ultrasound images were measured and averaged for each time of investigation. For a transformation to a usual clinical evaluation to describe the incisional site in the results, size of edema was transferred to degree of edema (slight (edema size 5–15 mm), moderate (>15 but ≤25 mm), or severe (>25 mm)). Presence and absence of sinus formation (defined as any fluid-filled subcutaneous cavity) was also assessed by ultrasound.

Between Day 10 and Week 8 horses were evaluated daily by clinical examination and any swelling at the incisional region and discharge from the incision site was recorded. However, no further standardized examinations were performed in this time period.

At Week 8 and 12 wound healing was re-evaluated by a clinical examination and ultrasound of the incision site. At this time point, an additional focus on abdominal herniation as well as any deviation of the ventral abdominal wall (minimal distorsions of the abdominal wall without palpable edges of a hernial ring) (Proudman, 2008) was undertaken.

Evidence of incisional infections was classified as yes in case of any positive microbiological culture in the drainage until Day 10. All other individuals were classified as no.

Statistical analysis relied on standard software^2^2IBM SPSS Statistics, IBM Corporation; Armonk, NY.. Data were checked for normality using the Shapiro–Wilk test. Categorical data for presence or absence of incisional infections was gathered in contingency tables and tested for dependency on suture pattern via Fisher’s exact test. Continuous data were calculated and expressed as mean ± standard deviation, unless otherwise stated. Comparison of duration of anaesthesia between groups were analysed by Mann–Whitney U-test. Dependency of edema size on group (CO vs. ID) or time was investigated by a linear mixed-effect model.

Level of significance for two-tailed tests was set at *p* < 0.05.

## Results

A total of 21 healthy geldings (5 warmbloods/5 ponies in group CO; 5 warmbloods/6 ponies in group ID) were included in this study. Overall mean age was 10.2 years (range, 5–13 years) in warmbloods and 8.4 years (range, 4–14 years) in ponies. Mean body weight was 592 kg (range, 477–665 kg) in warmbloods. In ponies, mean BW was 115 kg (83–186 kg).

In all animals coeliotomy was successfully completed and horses recovered without complications. Surgical time for celiotomy (time from incision to skin suture) did not differ between both groups, with a median value (± interquartile range) of 60 (±12.5) minutes in the CO group and 57.5 (±17.5) minutes in the ID group (*p* = 0.776). Excess gas in large colon and cecum had to be removed in two ponies (horses 1 and 7). In seven animals (horses 1, 3, 5, 7, 14, 20, and 21) mesocolic suturing was performed. None of the horses, which were mentioned above with any additional intra operative manipulations, developed incisional infection. During recovery, a self-adhesive drape dislodged in one animal (horse 20); the wound was disinfected and the drape was replaced. This horse did not experience an incisional infection.

Observed incisional complications are summarized in Table 2. Dehiscence did not occur.

**Table 2:** Breed, suture pattern, and incisional complications in each horse.

Horse no.	Breed	Suture pattern	Degree of edema on Day 6	Sinus formation (Day)	Surgical site infection (Day)	Hernia
**1**	P	CO	Severe	No	No	No
**2**	W	ID	Moderate	No	No	No
**3**	P	ID	Severe	No	No	No
**4**	W	ID	Slight	No	No	No
**5**	P	ID	Slight	No	No	No
**6**	W	CO	Severe	Yes (10)	Yes (4–12)	No
**7**	P	CO	Slight	No	No	No
**8**	W	ID	Severe	Yes (3 and 6)	No	No
**9**	P	ID	None	No	No	No
**10**	W	CO	Moderate	Yes (3 and 6)	Yes (6–12)	Yes
**11**	P	ID	Slight	No	No	No
**12**	W	CO	Moderate	No	No	No
**13**	P	CO	Slight	No	No	No
**14**	W	CO	Moderate	No	No	No
**15**	P	ID	Moderate	No	No	No
**16**	W	ID	Moderate	No	No	No
**17**	P	CO	None	No	No	No
**18**	P	CO	Slight	No	No	No
**19**	W	ID	Moderate	No	No	No
**20**	P	ID	Moderate	No	No	No
**21**	W	CO	Moderate	No	No	No

**Note:**

P, pony; W, warmblood; CO, continuous percutaneous pattern; ID, intradermal patterndegree of edema: slight (5–15 mm); moderate (>15 but ≤25 mm); severe (>25 mm).

Overall prevalence of incisional infection was 9.5%. With regard to suture pattern, incidence of surgical site infection was 20% and 0% in the CO and the ID group, respectively. However, this difference was not significant (*p* = 0.214).

Drainage, serosanguinous initially and later purulent, was documented in two warmbloods (horses 6 and 10, both in group CO). Each drainage was subsequently confirmed as incisional infections by microbiologic analysis of wound drainage (*Streptococcus equi* ssp. *zooepidemicus* in horse 6, *Streptococcus dysgalactiae* in horse 10), with both bacterial isolates being resistant to penicillin and gentamicin. Treatment in these cases consisted of intense physical massage with hydrotherapy. No local antibiotics were administered. Drainage persisted for 1 week and resolved without further treatment.

**Figure 1:**
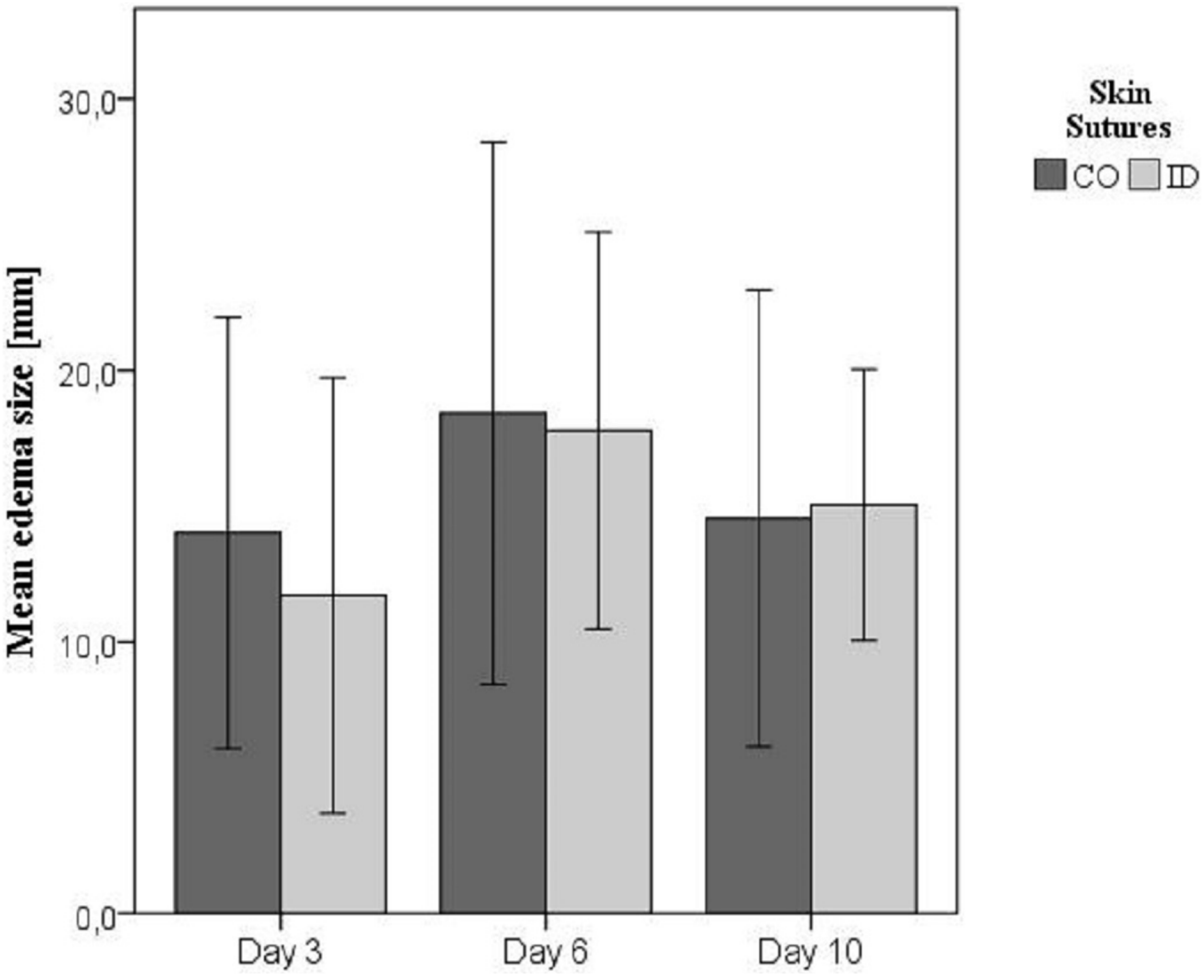
Mean edema size in millimeter (± standard deviation) on Days 3, 6, and 10. Edema size measured by transcutaneous ultrasound on Days 3, 6, and 10. Skin sutures: CO, continuous percutaneous pattern; ID, intradermal pattern.

Edema developed in all animals, with exception of two ponies (horses 9 and 17), occurring most frequently between Days 3 and 6 and peaking at Day 6. Values of edema size are presented in Fig. 1. Edema size was not dependent on time or group (*p* = 0.545 and *p* = 0.627, respectively).

Ultrasound confirmed sinus formation in three horses (2 warmbloods in group CO, 1 warmblood in group ID). Two of them developed the incisional drainage (horse 6 and horse 10, infection as proved by bacterial colonization; see above). In the horse from the ID group (horse 8), sinus formation resolved by Day 10, without any occurrence of incision site drainage.

Only one horse showed incisional herniation in Week 12. This horse suffered from incisional drainage previously. The hernia created a minimal distortion of the abdominal wall profile.

## Discussion

The purpose of this pilot study was to determine whether ID skin sutures could minimize the risk of incisional complications following ventral midline celiotomy in horses.

None of the horses subjected to ID suturing exhibited any signs of wound infection, which was not the case with closures by CO pattern. This effect might be of clinical importance, however, in this study it failed to have statistical significance.

A major advantage of this prospective randomized non-blinded experimental study is its use of a standardized protocol with regard to surgical technique (performed by a single surgeon), postoperative care and postoperative follow-up examinations by the same individual. Furthermore, the 12-week follow-up duration also enabled observation of late incisional complications, which has not been performed in such a setting before.

Intra- and postoperative management was standardized. In all cases, the incision was covered with self-adhesive drapes secured by skin staples for protection of the wound. Contamination during recovery is a common source of postoperative infection (Ingle-Fehr et al., 1997; Klohnen, 2009). By potentially exposing such horses to more incisional contamination than otherwise is encountered, poor quality of anaesthetic recovery bears an association with incisional infection (Freeman et al., 2012). Protective bandages may be used to lower rates of incisional infection (Smith et al., 2007; Tnibar et al., 2013), although this strategy confers no postoperative benefit according to Torfs et al. (2010). In our opinion, however, protecting the wound from contamination during recovery and early postoperative recuperation is of major importance. Self-adhesive drapes secured by skin staples were used as wound protection in our animals. The relation between quality of recovery and occurrence of surgical site infection was not investigated in this study.

In the current study, no perioperative antibiotics were given, due to the fact that use of antimicrobials in clean surgeries is controversial in both human and veterinary medicine and probably not necessary (Barie & Eachempati, 2005; Santschi, 2006). However, puncture of the large intestine needs to be classified as ‘clean-contaminated’ and, thereby, maybe necessitating a prophylactic use of antibiotics for 3 days, as it is common in clinical colic cases (Traub-Dargatz et al., 2002; Durward-Akhurst et al., 2013; Isgren et al., 2017). However, none of the ponies with gas removal from the intestine, developed surgical site infection. In the two horses, which suffered from abdominal wound infections, none of the antibiotics, which are routinely administered without any resistogram (penicillin and gentamicin) (Traub-Dargatz et al., 2002; Isgren et al., 2017), would have been effective in these cases. Consequently, antibiotic use should be well-considered and held to an absolute minimum depending on type of surgery and condition of the horses. In equine colic surgery, reduction of antibiotics to perioperative use only may be a reasonable approach.

Limitation of this pilot study is the design as a spin-off study on a homogenous cohort and, therefore, animal numbers, breed, and sex were determined by the global project. To avoid a bias by improved wound healing in ponies (Wilmink & Van Weeren, 2005), ponies and horses were equally distributed in ID or CO group.

The small sample size in combination with analysis of categorical data (yes/no) have mainly contributed to a type II error and, therefore, this study failed to give any statistical significance. A post hoc power analysis for a reduction of incisional infection in one group by 20%, revealed that per group 45 horses would have been necessary to obtain significant differences between both groups. Therefore, larger studies with inclusion of equine patients are warranted to confirm statistically significant differences in skin suture pattern.

Another limitation of this study was the inability to blind the postoperative investigator due to the obvious existence of skin sutures. However, examinations of the incision site were categorized qualitatively (yes/no) or quantitatively in case of edema size. Therefore, biased results were reduced.

In this study, closure of the laparotomy wounds involved four layers: peritoneum, linea alba, subcutis, and skin. While many surgeons may omit peritoneal closure, this step is critical in our opinion, helping to minimize problems in wound healing. In doing so, efflux of peritoneal fluid or sterile washings (used to flush the abdominal cavity during surgery) into the wound and subcutaneous swelling are prevented. To avoid any risk of peritoneal rupture while suturing, peritoneum, and linea alba may be closed in a fractional way. However, even the latest editions of textbooks on equine surgery still suggest that suturing the peritoneum encourages the formation of abdominal adhesions (Freeman, 2008). This is in reference to a publication by Swanwick & Milne (1973) examining the aggressive use peritoneal sutures under experimental conditions. Although peritoneal adhesions occurred at a 50% rate with use of polyester suture, the corresponding rate for unsutured peritoneum was 27.2%. It is our contention that these conclusions warrant a closer look. In another study, Huskamp also maintains that suturing of the peritoneum is an essential step in the closure of laparotomy wounds (Huskamp, 1982). This opinion is supported by studies comparing rates of infected incisions after laparotomy for colic surgery in horses, in which a significant reduction in wound healing complications were detected when peritoneal suturing was performed compared to unsutured peritoneum (Torfs et al., 2010; Scharner et al., 2017).

In the current study, subcutaneous suturing was performed, as this has shown to reduce the likelihood of developing incisional complications (Smith et al., 2007). This is in accordance with Halsted’s principles, particularly the need to avoid dead space. Moreover, the time or effort saved by abandoning subcutaneous closure, when using a two-layer closure technique, is negligible, relative to total operative duration. For subcutaneous suturing, we used Vicryl™ Plus, an antibacterial-coated suture proven to prevent in vitro and in vivo colonization by *Staphylococcus aureus*, multiresistant *Staphylococcus aureus*, *Staphylococcus epidermidis*, and *E. coli*, thus potentially reducing surgical site infections (Rothenburger et al., 2002; Storch, Rothenburger & Jacinto, 2004; Edmiston et al., 2006; Marco et al., 2007). Still, the efficacy of such material is subject to debate, having failed to reduce the likelihood of incisional complications in the current study and in another study (Bischofberger et al., 2010).

Skin closure traditionally is achieved by non-absorbable suture in continuous or interrupted pattern. This method is represented by the CO group. Conceptually, the apposition of skin edges (and therefore sealing of the wound) attained by suturing is superior to that of stapling. This might be an explanation for increased risk of incisional infection when using staples for skin closure (Torfs et al., 2010). In humans, ID suturing is often selected for aesthetic reasons, but also yielding good results in terms of incisional infection (Johnson, Young & Reilly, 2006), potentially due to tightness of wound edges, and avoidance of a percutaneous suture canal. A similar finding in horses were published by Colbath et al. (2014), in which a significantly reduced incidence of incisional drainage was found in accordance with a subcuticular closure compared to a percutaneous skin closure. Similarly, in the present study none of the horses subjected to cutaneous ID suturing exhibited any signs of wound infection. However, the present study failed to show any significant difference between groups with regard to surgical site infection. When comparing ID and CO suturing, it should be taken into account, that in both groups different suture materials have been used for skin closure (see Table 1) and might have influenced this result.

Incisional edema was the most frequent finding in wound healing in our study horses. This is in accordance with other studies, reporting a prevalence of edema of 70–90% (Santschi, 2006; Smith et al., 2007; Bischofberger et al., 2010; Anderson, Bracamonte & Hendrick, 2014), which is not regarded as a postoperative complication in many studies. However, if severe and persistent, an abnormal healing of the incision is likely (Gibson et al., 1989). Furthermore, some studies show a strong correlation between severe wound edema and subsequent surgical site infection (Coomer et al., 2007; Scharner et al., 2017). This was not shown in the present cohort, possibly related to the lack of sufficient power of the study.

The definition of incisional infection, suppuration and drainage varies greatly in different studies, making comparisons difficult. By some authors, any drainage is indicative of infection (Ingle-Fehr et al., 1997; Torfs et al., 2010; Freeman et al., 2012). Other studies differentiate between drainage and infection (Smith et al., 2007; Colbath et al., 2014; Darnaud et al., 2016; Isgren et al., 2017) and partially consider serosanguinous wound discharge as a normal accompaniment of recovery from anaesthesia. However, we consider this as a lack of tight abdominal wound closure, not seen in our population of animals.

Dehiscence did not occur in our research horses. Although the reported incidence is low (1.1–9%), incisional dehiscence of the equine abdomen is a catastrophic and potentially fatal event, especially if linea alba is involved (Kobluk et al., 1989; Smith et al., 2007; Bischofberger et al., 2010; Anderson, Bracamonte & Hendrick, 2014; Anderson et al., 2015).

Sinus formation was diagnosed via ultrasound. The latter is essential for detecting this condition (Wilson et al., 1989; Trostle & Hendrickson, 1995; Chism et al., 2000; Lippold, 2001). In our animal population, sinuses developed in three horses (14.3%); but progression to incisional infection occurred in only two of them. In contrast, Wilson et al. (1989) observed a proportionately high rate (71.4%) of sinus formation in their study group.

Incisional hernia did occur in one of the horses we studied (4.8%). This occurred following a previous incisional infection. Published incisional hernia rates range from 3.2% to 17% (Gibson et al., 1989; Kobluk et al., 1989; Phillips & Walmsley, 1993; Wilson, Baker & Boero, 1995; Honnas & Cohen, 1997; Anderson et al., 2015), which is in accordance with our findings. Overall, our observed prevalence of incisional infection was low (9.5%), based on healthy horses in an experimental setting. However, haemodynamic conditions might be altered by LPS-infusion prior surgery leading to systemically compromised individuals, similarly to colic horses. Despite a lack of abnormalities in the clinical evaluation prior to anaesthesia in the study horses, the alterations of haematocrit, platelet function, white blood cells and cardiac output by LPS-infusion was not investigated in this part of the study. Therefore, abnormalities could have been present 15 h after LPS-administration, as shown in a study in foals with experimental endotoxemia (Wong et al., 2013). Moreover, it should be taken into account that no analgesic has been administered after the LPS-challenge before surgery in our study. With regard to the intestinal handling and tissue sampling that were done, with some intra-abdominal bleeding, manipulations were comparable to relatively simple colic surgery (without enterotomy or resection). Moreover, it has been shown, that opening of the bowel did not influence occurrence of incisional infection (Gibson et al., 1989; Kobluk et al., 1989; Phillips & Walmsley, 1993; Wilson, Baker & Boero, 1995; Coomer et al., 2007; Bischofberger et al., 2010).

It is worth mentioning than none of the ponies developed an incisional infection. This might be an effect of reduced body weight (Wilson, Baker & Boero, 1995; Darnaud et al., 2016) or a breed specificity, which is associated with better wound healing (Wilmink & Van Weeren, 2005). However, due to the small sample size and the low incidence of surgical site infection, a comparison between ponies and horses was not possible.

## Conclusion

In conclusion, both CO and ID suture patterns are applicable for skin closure following ventral median celiotomy in horses. None of the animals in the continuous ID group developed surgical site infections, even without use of antibiotics. However, further studies are necessary to prove this beneficial effect in clinical cases.

## Supplemental Information

10.7717/peerj.5772/supp-1 Supplemental Information 1 Edema size measured at Day 3, 6 and 10.

## Additional Information and Declarations

### Data Availability

The following information was supplied regarding data availability: The raw data are included in a Supplemental File.
