# Supplementary material for: Comparison of incisional complications between skin closures using a simple continuous or intradermal pattern: a pilot study in horses undergoing ventral median celiotomy
Source: PeerJ. 2018 Nov 9;6:e5772. doi: 10.7717/peerj.5772 (PMC6231425; doi:10.7717/peerj.5772)
Supplement: Supplemental Information 1 [file peerj-06-5772-s001.docx]

|  |  | Edema Size in mm | | |
| --- | --- | --- | --- | --- |
| Horse ID | suture technique | Day 3 | Day 6 | Day 10 |
| 1 | CO | 25.5 | 32.2 | 20.8 |
| 2 | ID | 10 | 18.6 | 10 |
| 3 | ID | 30.5 | 30 | 24.9 |
| 4 | ID | 10 | 14 | 12 |
| 5 | ID | 14.4 | 13 | 13 |
| 6 | CO | 7.7 | 34.1 | 24.3 |
| 7 | CO | 12.3 | 5.3 | 6.7 |
| 8 | ID | 4.8 | 27.2 | 16 |
| 9 | ID | 3.5 | 3.6 | 4.1 |
| 10 | CO | 27.3 | 19.9 | 19.8 |
| 11 | ID | 4.6 | 14.8 | 12.6 |
| 12 | CO | 4.5 | 19.6 | 19.4 |
| 13 | CO | 13.1 | 14.6 | 7.6 |
| 14 | CO | 13.9 | 16.1 | 15.6 |
| 15 | ID | 9.4 | 17.6 | 19.9 |
| 16 | ID | 6.4 | 15.7 | 14.1 |
| 17 | CO | 4 | 4.4 | 4.2 |
| 19 | CO | 11 | 13.2 | 5 |
| 20 | ID | 15.2 | 16.7 | 13.1 |
| 21 | ID | 19.9 | 24.4 | 21.2 |
| 22 | CO | 19.8 | 24.9 | 24 |
